# In the national epidemiological bulletins – a selection from current issues

**DOI:** 10.2807/1560-7917.ES.2017.22.13.30495

**Published:** 2017-03-30

**Authors:** Eurosurveillance editorial team

**Affiliations:** 1 European Centre for Disease Prevention and Control (ECDC), Stockholm, Sweden

**Keywords:** infectious diseases, public health, Europe

**Healthcare-associated infections and antibiotic resistance**

 

Point Prevalence Survey of Hospital Acquired Infections and Antimicrobial Use in Ireland 2017

Epi-Insight 2017;18(3)

March 2017, Ireland

http://ndsc.newsweaver.ie/epiinsight/1kxgb6ek1nx?a=1&p=51521198&t=17517774

 

Adaptation of isolation measures in case of livestock-associated multiresistant *Staphylococcus aureus* (LA-MRSA) leads to better patient care

Infectieziekten Bulletin 2017; 28(2)

24 February 2017, the Netherlands

http://www.rivm.nl/dsresource?objectid=ab0ce6b4-98d2-4a37-a5e0-be92adfab4f8&type=pdf&disposition=inline

 

Multiresistant *Staphylococcus aureus* on pig farms

Infectieziekten Bulletin 2017; 28(2)

24 February 2017, the Netherlands

http://www.rivm.nl/dsresource?objectid=fcaeeac9-5fde-4d54-87d9-e521780685e5&type=pdf&disposition=inline

 

Genetic exchange between multiresistant *Staphylococcus aureus*

Infectieziekten Bulletin 2017; 28(2)

24 February 2017, the Netherlands

http://www.rivm.nl/dsresource?objectid=f6a62f77-9997-4930-8dca-febd780fe65c&type=pdf&disposition=inline

 

Antibiotic resistance in gastrointestinal bacteria

Infectieziekten Bulletin 2017; 28(1)

24 January 2017, the Netherlands

http://www.rivm.nl/dsresource?objectid=9d23345f-3449-45f7-9934-021076cf3bd9&type=pdf&disposition=inline

 

National Annual Antimicrobial Point Prevalence Survey 2016

Epi-Insight 2016;17(12)

December 2016, Ireland

http://ndsc.newsweaver.ie/epiinsight/kgky01jxfir?a=1&p=51164834&t=17517774

 

**Food- and waterborne diseases**

 

Authorities are investigating new cases of EHEC infection

Folkhälsomyndigheten website

13 March 2017, Sweden

https://www.folkhalsomyndigheten.se/nyheter-och-press/nyhetsarkiv/2017/mars/myndigheter-utreder-nya-fall-av-ehec-infektion/

 

Occurrence of *Clostridium difficile* ribotype 176 in Germany

Epidemiologisches Bulletin 10, 2017

9 March 2017, Germany

https://www.rki.de/DE/Content/Infekt/EpidBull/Archiv/2017/Ausgaben/10_17.pdf?__blob=publicationFile

 

Need to reduce *Campylobacter* in chicken meat

Folkhälsomyndigheten website

10 February 2017, Sweden

https://www.folkhalsomyndigheten.se/nyheter-och-press/nyhetsarkiv/2017/februari/campylobacter-pa-kycklingkott-behover-minska/

 

Annual summary of *Salmonella* and *Campylobacter* infections

HPS Weekly Report 2017; 51(05)

7 February 2017, Scotland

http://www.hps.scot.nhs.uk/documents/ewr/pdf2017/1705.pdf

 

A cluster of *Campylobacter* infection among a group of youngsters

Infectieziekten Bulletin 2017; 28(1)

24 January 2017, the Netherlands

http://www.rivm.nl/dsresource?objectid=71b5ca01-ba17-42f9-9d27-12460ee4b38e&type=pdf&disposition=inline

 

Surveillance of *Listeria monocytogenes* in the Netherlands in 2015

Infectieziekten Bulletin 2017; 28(1)

24 January 2017, the Netherlands

http://www.rivm.nl/dsresource?objectid=47e55962-7d01-4ae6-8b2d-edba4f88a40e&type=pdf&disposition=inline

**

*Salmonella* typhimurium infection in infants after a trip to a farm

Vlaams Infectieziektebulletin; 4/2016

December 2016, Belgium

https://www.zorg-en-gezondheid.be/sites/default/files/atoms/files/2016-4-salmon-T-CH.%20Lambrechts.pdf

 

**Hepatitides**

 

Acute and chronic Hepatitis C, 2016

EPI-NEWS 11, 2017

15 March 2017, Denmark

http://www.ssi.dk/Aktuelt/Nyhedsbreve/EPI-NYT/2017/Uge%2011%20-%202017.aspx

 

Acute and chronic Hepatitis B 2016

EPI-NEWS 9, 2017

1 March 2017, Denmark

http://www.ssi.dk/Aktuelt/Nyhedsbreve/EPI-NYT/2017/Uge%209%20-%202017.aspx

 

Hepatitis B infection in Scotland: 2015

HPS Weekly Report 2017; 51(02)

17 January 2017, Scotland

http://www.hps.scot.nhs.uk/documents/ewr/pdf2017/1702.pdf

 

**Vaccine-preventable diseases**

 

Invasive meningococcal disease (laboratory reports in England): England: October to December 2016

Health Protection Report; 11(8)

24 February 2017, United Kingdom

https://www.gov.uk/government/uploads/system/uploads/attachment_data/file/594800/hpr0817_imd.pdf

 

Pertussis vaccination programme for pregnant women update: vaccine coverage in England, October to December 2016

Health Protection Report; 11(8)

24 February 2017, United Kingdom

https://www.gov.uk/government/uploads/system/uploads/attachment_data/file/594799/hpr0817_prntl-prtsss-VC.pdf

 

Examination of a pertussis outbreak in children with high vaccination rates in Kiel, October 2015-March 2016

Epidemiologisches Bulletin 6, 2017

9 February 2017, Germany

https://www.rki.de/DE/Content/Infekt/EpidBull/Archiv/2017/Ausgaben/06_17.pdf?__blob=publicationFile

 

Increase in the incidence of invasive meningococcal disease caused by group W135

EPI-NEWS 6, 2017

8 February 2017, Denmark

http://www.ssi.dk/Aktuelt/Nyhedsbreve/EPI-NYT/2017/Uge%206%20-%202017.aspx

 

Shingles vaccine coverage report, England, September to November 2016

Health Protection Report; 11(4)

27 January 2017, United Kingdom

https://www.gov.uk/government/uploads/system/uploads/attachment_data/file/586837/hpr0417_shngls.pdf

 

Sudden increase of invasive meningococcal disease serogroup W in 2015 and 2016

Infectieziekten Bulletin 2017; 28(1)

24 January 2017, the Netherlands

http://www.rivm.nl/dsresource?objectid=9fcfee07-c3dc-4af8-9437-7e328a66469c&type=pdf&disposition=inline

 

Measles, mumps and rubella and childhood vaccine uptake

HPS Weekly Report 2017; 51(01)

10 January 2017, Scotland

http://www.hps.scot.nhs.uk/documents/ewr/pdf2017/1701.pdf

 

New childhood immunisation schedule

Epi-Insight 2017;18(1)

January 2017, Ireland

http://ndsc.newsweaver.ie/epiinsight/uc4nexz6bh2?a=1&p=51284091&t=17517774

 

Cases of measles in North Zealand

EPI-NEWS 1/2, 2017

11 January 2017, Denmark

http://www.ssi.dk/Aktuelt/Nyhedsbreve/EPI-NYT/2017/Uge%201-2%20-%202017.aspx

 

Laboratory confirmed cases of pertussis reported to the enhanced pertussis surveillance programme in England during July to September 2016

Health Protection Report; 10(44)

16 December 2016, United Kingdom

https://www.gov.uk/government/uploads/system/uploads/attachment_data/file/578804/hpr4416_prtsss.pdf

 

Invasive meningococcal disease (laboratory reports in England): England: July to September 2016

Health Protection Report; 10(44)

16 December 2016, United Kingdom

https://www.gov.uk/government/uploads/system/uploads/attachment_data/file/578806/hpr4416_imd.pdf

 

National measles outbreak in Ireland declared over

Epi-Insight 2016;17(12)

December 2016, Ireland

http://ndsc.newsweaver.ie/epiinsight/y461qge49cz?a=1&p=51143038&t=17517774

 

**Respiratory diseases**

**

*Legionella pneumonia* and Pontiac fever at a campsite in Croatia

Infectieziekten Bulletin 2017; 28(3)

23 March 2017, the Netherlands

http://www.rivm.nl/dsresource?objectid=b2fe27ea-1c7a-4378-9be2-8d44f0262d04&type=pdf&disposition=inline

 

Tuberculosis epidemiology in France in 2015. Impact of the suspension of BCG mandatory vaccination on child tuberculosis, 2007-2015

Bulletin épidémiologique hebdomadaire, 7

21 March 2017, France

http://invs.santepubliquefrance.fr/beh/2017/7/pdf/2017_7.pdf

 

Anti-tuberculosis drug resistance in France in 2014-2015

Bulletin épidémiologique hebdomadaire, 7

21 March 2017, France

http://invs.santepubliquefrance.fr/beh/2017/7/pdf/2017_7.pdf

 

Status of influenza season 2016/17

EPI-NEWS 5, 2017

1 February 2017, Denmark

http://www.ssi.dk/Aktuelt/Nyhedsbreve/EPI-NYT/2017/Uge%205%20-%202017.aspx

 

Influenza vaccine uptake among healthcare workers – interim survey results mid season 2016-2017

Epi-Insight 2017;18(1)

January 2017, Ireland

http://ndsc.newsweaver.ie/epiinsight/1p50tjai5xn?a=1&p=51284092&t=17517774

 

Respiratory bacteria quarterly report. Quarter three: 1 July to 31 September 2016

HPS Weekly Report 2016; 50(50)

13 December 2016, Scotland

http://www.hps.scot.nhs.uk/documents/ewr/pdf2016/1650.pdf

 

**Sexually transmitted diseases**

 

ANSWER. HIV infection and AIDS: Quarterly report to 31 December 2016

HPS Weekly Report 2017; 51(11)

21 March 2017, Scotland

http://www.hps.scot.nhs.uk/documents/ewr/pdf2017/1711.pdf

 

New HIV preventive drug’s acceptance and constraints of African and Caribbean population living in Ile-de-France: the use of Truvada® in pre-exposure prophylaxis (PrEP). An exploratory survey

Bulletin épidémiologique hebdomadaire, 6

7 March 2017, France

http://invs.santepubliquefrance.fr/beh/2017/6/pdf/2017_6.pdf

 

Increase in gonorrhoea cases in the South-East: heterosexual transmission a feature

Epi-Insight 2017;18(3)

March 2017, Ireland

http://ndsc.newsweaver.ie/epiinsight/83681u1ih1x?a=1&p=51521199&t=17517774

 

Recent outbreak of ciprofloxacin-resistant *Shigella* among MSM in Dublin

Epi-Insight 2017;18(2)

February 2017, Ireland

http://ndsc.newsweaver.ie/epiinsight/y9rvan81q6z10gkzp9yxn5?a=2&p=51399875&t=17517804

 

Persons living with HIV in Italy: data from the second national survey

Not Ist Super Sanità 2017;30(1)

January 2017, Italy

http://www.iss.it/binary/publ/cont/ONLINE_GENNAIO.pdf

 

Further increase in syphilis infections in men who have sex with men

Epidemiologisches Bulletin 50, 2016

19 December 2016, Germany

http://www.rki.de/DE/Content/Infekt/EpidBull/Archiv/2016/Ausgaben/50_16.pdf?__blob=publicationFile

 

**Zoonoses and vector-borne diseases**

 

Malaria and illegal gold mining in French Guiana: a major public health challenge

Bulletin épidémiologique hebdomadaire, 6

7 March 2017, France

http://invs.santepubliquefrance.fr/beh/2017/6/pdf/2017_6.pdf

 

Creutzfeldt-Jakob disease (CJD) biannual update (February 2017)

Health Protection Report; 11(6)

10 February 2017, United Kingdom

https://www.gov.uk/government/uploads/system/uploads/attachment_data/file/591266/hpr0617_cjd.pdf

 

Several cases of psittacosis among people who cleaned bird feeders

Folkhälsomyndigheten website

26 January 2017, Sweden

https://www.folkhalsomyndigheten.se/nyheter-och-press/nyhetsarkiv/2017/januari/flera-fall-av-papegojsjuka-bland-personer-som-rengjort-fagelbord/

 

**Other**

 

Increase in reporting of ‘polio-like’ diseases in 2016

Epidemiologisches Bulletin 9, 2017

2 March 2017, Germany

https://www.rki.de/DE/Content/Infekt/EpidBull/Archiv/2017/Ausgaben/09_17.pdf?__blob=publicationFile

 

How long is scabies crustosa contagious in a deceased patient?

Infectieziekten Bulletin 2017; 28(1)

24 January 2017, the Netherlands

http://www.rivm.nl/dsresource?objectid=a0d3b667-4f75-4b94-ba63-01f4fafec1d9&type=pdf&disposition=inline

 

Antenatal screening for infectious diseases in England: summary report for 2015

Health Protection Report; 11(2)

13 January 2017, United Kingdom

https://www.gov.uk/government/uploads/system/uploads/attachment_data/file/583576/hpr0217_naism.pdf

